# Complement activation in the injured central nervous system: *another dual-edged sword?*

**DOI:** 10.1186/1742-2094-9-137

**Published:** 2012-06-21

**Authors:** Faith H Brennan, Aileen J Anderson, Stephen M Taylor, Trent M Woodruff, Marc J Ruitenberg

**Affiliations:** 1 The University of Queensland, School of Biomedical Sciences, St Lucia, Brisbane, QLD, 4072, Australia; 2 Anatomy & Neurobiology, University of California, Irvine, USA; 3 The Queensland Brain Institute, The University of Queensland, Brisbane, QLD, 4072, Australia

## Abstract

The complement system, a major component of the innate immune system, is becoming increasingly recognised as a key participant in physiology and disease. The awareness that immunological mediators support various aspects of both normal central nervous system (CNS) function and pathology has led to a renaissance of complement research in neuroscience. Various studies have revealed particularly novel findings on the wide-ranging involvement of complement in neural development, synapse elimination and maturation of neural networks, as well as the progression of pathology in a range of chronic neurodegenerative disorders, and more recently, neurotraumatic events, where rapid disruption of neuronal homeostasis potently triggers complement activation. The purpose of this review is to summarise recent findings on complement activation and acquired brain or spinal cord injury, i.e. ischaemic-reperfusion injury or stroke, traumatic brain injury (TBI) and spinal cord injury (SCI), highlighting the potential for complement-targeted therapeutics to alleviate the devastating consequences of these neurological conditions.

## Introduction

Injury to the central nervous system (CNS) elicits a complex series of pathophysiological events, including ischaemia, excitotoxicity and inflammation. All of these factors adversely affect the integrity of spared neurons and thus accentuate tissue damage beyond the initial site of trauma. The cellular immune response in particular has received much attention as a key mediator of secondary injury, and strategies to manipulate the activation and recruitment of neutrophils [1-5], monocytes and macrophages [6-9], and lymphocytes [10-12] after trauma have all been investigated, with the ultimate goal being to improve functional outcomes (reviewed in [13]).

Several recent studies have, however, put activation of the innate immune complement system into the spotlight as a perhaps sometimes-overlooked but potent mediator of secondary pathology [14-16]. The particular aim of this review is to summarise current knowledge and understanding of complement activation in the injured CNS, specifically in relation to post-traumatic neuroinflammatory events and associated secondary damage. Several other recent reviews have already provided a comprehensive overview of the role of complement in CNS development and chronic neurodegenerative disorders [17-19].

### The complement system: an introduction and effector mechanisms

**Figure 1:**
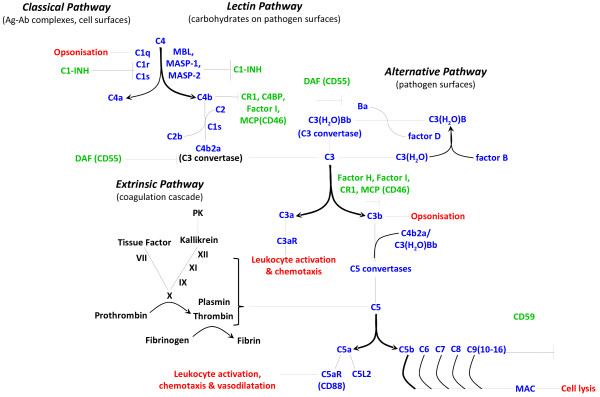
**Common pathways for complement activation.** Recognition of antigen-antibody complexes by C1q initiates the *‘classical pathway’*. Binding of carbohydrate antigens by mannose-binding lectin (MBL) or MBL-associated serine proteases (MASPs) initiates the *‘lectin pathway’*. Both pathways lead to the formation of the C3 convertase, C4b2a. Complement activation through the *‘alternative pathway’* involves the spontaneous hydrolysis of plasma C3, generating a second C3 convertase, C3(H_2_O)Bb*.* Proteolysis of C3 then leads to production of the C3b fragment, which binds to C3 convertases to generate C5 convertases. After the cleavage of C5, the C5b fragment binds C6-C9 to generate the membrane attack complex (MAC). The coagulation cascade leads to complement activation via the *‘extrinsic pathway’*; this route does not depend on the presence of C3 convertases. Anaphylatoxins C5a, C3a and C4a are generated through cleavage of C5, C3 and C4, respectively. Soluble and membrane-bound negative regulators of complement and their site of action are indicated in *green*. The functional significance of certain activation steps is shown in *red*.

The predominant site of peripheral complement protein synthesis is the liver, where hepatocytes constantly produce and replenish circulating complement factors [20]. Activation of these circulating complement proteins in response to an injurious or infectious challenge results in a self-amplifying cascade of proteolytic reactions through any one of four major identified pathways (Figure 1).

The *classical pathway* for complement activation is initiated by the binding of the recognition molecule C1q to pathogen antigens, C-reactive protein bound to bacterial polysaccharides or antigen-antibody complexes [21]. It is of interest to note in this context that pathogen opsonisation and antibody ligation by C1q also provide a bridge to activation of the adaptive immune system, which includes an enhancement of antigen retention in lymphoid tissues, a decrease in the B cell activation threshold and increased memory B cell survival [22-24]. T cell proliferation, differentiation, activation and antigen-presenting cell (APC) function can also be significantly influenced by complement [25,26]. The *lectin pathway* for complement activation involves the recognition of pathogen carbohydrate antigens by mannose-binding lectin-associated serine proteins (MASP-1 and MASP-2) [27] and the ficolins [28]. The *alternative pathway* of complement activation is initiated by spontaneous hydrolysis of complement component C3 in plasma, and the binding of factor B and D to C3(H_2_O) [29]. All of the three aforementioned activation routes lead to the formation of C3 convertases and thus converge at this level.

C3 convertases cleave the parental C3 molecule into two fragments, the larger C3b molecule and the smaller anaphylatoxin C3a. The C3b fragment opsonises pathogen-associated molecular patterns (PAMPs), which are small, conserved molecular motifs that are shared by classes of microbes and recognised by host cell pattern recognition receptors (PRRs), such as Toll-like receptors (TLRs) [30]. C3b opsonises altered-self ligands, immune complexes and/or dead cells as well, which ultimately enhances their recognition and rapid phagocytosis by scavenging leukocytes that bear C3b receptors. The C3b fragment can also bind the C3 convertase, which leads to the formation of a C5 convertase and the subsequent cleavage of the parental C5 protein into C5b and the anaphylatoxin C5a. The amplification cascade then culminates in the association of C5b with C6, C7 and C8, which induces the polymerisation of 10–16 C9 molecules in order to assemble a transmembrane pore called the terminal ‘membrane attack complex’ (MAC), with subsequent lysis of the targeted pathogens or abnormal host cells as a result [31]. Importantly, components of the blood clotting and fibrinolysis pathways, as well as other cell-derived serine proteases, can also directly cleave and activate C3 and C5 proteins, and thus initiate the formation of complement end products, independent of the C3 and C5 convertases, a process that is now referred to as the *extrinsic pathway*[32-34].

As indicated above, the cleavage of C3 and C5 also leads to the generation of two smaller activation fragments that do not directly contribute to MAC formation, the so-called anaphylatoxins. The small cleavage product of the parental C3 protein, the anaphylatoxin C3a, is a local mediator of inflammation that signals through its G-protein coupled receptor, C3aR [35]. The anaphylatoxin C5a, generated from C5, is one of the most potent pro-inflammatory peptides known. It can act as a phagocyte chemoattractant, and promote vascular permeability, platelet and leukocyte activation through, for example, upregulation of the leukocyte adhesion molecules necessary for transendothelial extravasation to sites of infection or injury. It also induces production of proinflammatory cytokines, chemokines, leukotrienes, prostaglandins, oxidative burst and degranulation (reviewed in [31,36]). Similar to C3a, the C5a molecule signals through a G-protein coupled receptor, which is known as C5aR or CD88 [37]. A second receptor for C5a, known as C5L2, has been reported [38], but its functional significance still remains controversial; indeed, it could have multiple roles in different species, organs and pathophysiological states [39-42].

### Physiological functions for complement in the CNS

Although circulating complement proteins in blood plasma do not normally have access to the CNS because of the blood–brain and blood-spinal cord barriers (BBB and BSB, respectively), several studies have demonstrated that virtually all of the components of complement can be synthesized within the CNS [18,19,43]. Accordingly, various non-immune physiological roles for complement have been identified, including synaptic remodelling during development, cell survival and neurogenesis.

A seminal study by Stevens and colleagues [44] showed that components of the classical complement pathway are key mediators of synapse elimination in the developing retinogeniculate pathway in mice. Early in development, retinal ganglion cells (RGCs) from both eyes extend excessive, overlapping projections into the dorsal lateral geniculate nucleus (dLGN) of the thalamus. Weaker synaptic arborisations are then eliminated and more active connections strengthened, resulting in the adult pattern of segregated eye layers by postnatal day 20 [45]. This process is coordinated by astrocyte-driven deposition of C1q and C3 on immature or weaker RGC synapses, which then tags them for removal, most likely by activated microglia. RGC axons in C1q^−/−^ and C3^−/−^ mice have indeed a higher degree of overlap during and after the remodelling process, resulting in the persistent retention of excessive retinal innervations of lateral geniculate neurons [44]. The supernumerary inputs retained in C1q^−/−^ and C3^−/−^ neurons were shown to be immature or dysfunctional as judged by the weak magnitude of the glutamate-mediated currents carried by their AMPA receptors [44]. Interestingly, the failure to prune excessive excitatory synapses during development has been positively correlated to enhanced synaptic connectivity and epileptogenesis in C1q^−/−^ mice compared to wild-type (WT) controls [46]. Our groups have also previously shown localized expression of CD88, the C5a anaphylatoxin receptor, on presynaptic terminals of mossy fibres within the CA3 region of the adult rat hippocampus [47]; it remains to be determined whether physiological C5a signalling here could also be involved in synaptic plasticity or whether it serves different and yet unknown functions in this structure.

In addition to a role in synaptic plasticity, complement proteins may play an important role in neuroprotection in the CNS. In the absence of other complement components, C1q has been shown to increase neuronal survival and arborisation [48]. These effects are mediated via the upregulation of genes associated with cytoskeleton function (syntaxin-3), cholesterol/lipid metabolism (CH25H, INSIG2) and neurotrophic factors (NGF, NT-3, NTN1) [49].

A functional role for the MAC has also been demonstrated. At sublytic concentrations, the MAC can be endocytosed by oligodendrocytes and cause them to re-enter the cell cycle [50]. Sublytic MAC can also reduce apoptotic cell death by increasing synthesis of Bcl-2 and inhibition of caspase-3 activation and caspase-8 processing and upregulating FLIP [51,52].

Lastly, a physiological role for the anaphylatoxins C3a and C5a within the adult murine CNS has emerged, specifically an involvement in cell survival and neurogenesis. In mixed cultures of neurons and astrocytes, C3a protected neurons against NMDA-induced excitotoxicity in a dose- and astrocyte-dependent manner [53]. The neuroprotective action of C3a signalling could be mediated through the induction of nerve growth factor (NGF) expression in microglia [54,55] as well as astrocytes [55]. C5a exposure also causes an upregulation of NGF mRNA expression in astrocytes [55], with similar neuroprotective effects against glutamate-mediated neuronal excitotoxicity. The neuroprotective effects of anaphylatoxins against glutamate-induced excitotoxicity were shown to be mediated via MAPK-dependent inhibition of caspase-3 [56,57], regulation of glutamate receptor subunit 2 (GluR2) expression [58], and increased glial expression of the glutamate transporter GLT-1, which enhances the removal of extracellular glutamate [59]. Administration of C5a in vivo was also reported to protect against kainic acid-induced neuronal apoptosis [56]. With regards to a role for anaphylatoxins in neurogenesis, the respective receptors for C3a and C5a, C3aR and CD88 were shown to be expressed on neural progenitor cells as well as immature neurons [60]. Mice treated with a non-specific C3aR antagonist (SB290157) displayed decreased formation of new neurons in areas of adult neurogenesis [60]. The cellular or humoral source of C3a and the mechanism via which C3aR activation influences the creation and/or survival of new neurons remain to be elucidated. However, in the post-natal developing cerebellum, C3aR and CD88 expressions are known to increase during granular cell maturation [61]. Subdural injection of a non-specific CD88 agonist (MAP-C5a) into the cerebellum of young rats increased the proliferation of immature granule neurons, resulting in an enlarged external granule cell layer [62,63]. This effect could be reversed through concurrent administration of PMX53, a specific CD88 antagonist [63,64]. A C3a agonist (MAP-C3a), on the other hand, decreased the thickness of the EGL whilst increasing the thickness of the internal granule cell layer. Video microscopy revealed that C3a accelerated the migration process of granule cells from the EGL to the internal granule cell layer [63].

### Complement and CNS disorders

Various regulators normally finely tune the complement activation repertoire so that healthy host tissue is discriminated and self-harm is avoided. However, a disturbed balance between activation and regulation can induce self-attack, and excessive or inappropriate complement activation has been implicated in the pathogenesis of numerous autoimmune, ischaemic and vascular diseases [31,65]. Complement deregulation has also been proposed in a myriad of CNS inflammatory pathologies and, as a result, complement-targeted therapeutics are increasingly emerging into the spotlight of drug discovery endeavours for various chronic neurodegenerative diseases, including multiple sclerosis [66-74], Alzheimer’s disease [75-78], Huntington's disease [79,80], Parkinson’s disease [81,82] and motor neuron disease [83-86]. Although breakdown of the BBB and BSB does not occur until very late in most neurodegenerative pathologies, a prominent role for complement is perhaps not surprising when one considers again that the CNS can endogenously synthesise virtually all components of the complement system under appropriate stimuli [87]. Furthermore, neurons and oligodendrocytes express only low levels of the surface complement regulatory protein decay activating factor (DAF/CD55) and membrane cofactor protein (MCP/CD46), which renders them particularly vulnerable to complement-associated death [88,89].

**Figure 2:**
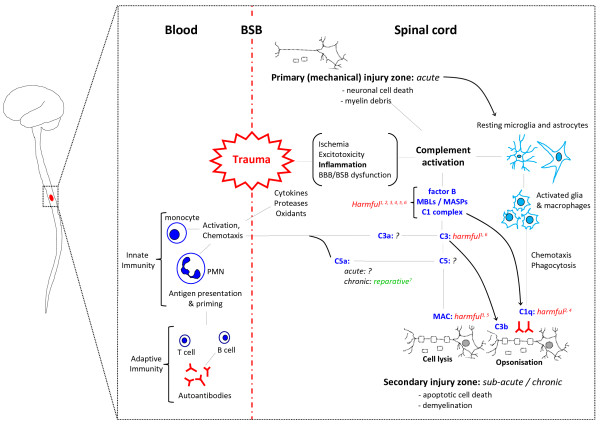
**Schematic diagram showing the current understanding of the role of complement activation in the pathophysiology associated with traumatic spinal cord injury (SCI).** Mechanical damage to the spinal cord causes neuronal cell death and disruption of the blood-spinal cord barrier (*BSB*). This primary damage triggers a potent inflammatory response and initiates complement activation. Although complement activation may aid the clearance of cellular debris through opsonisation, it is also known to potentiate injury beyond the site of trauma through e.g. the opsonins C1q, C3b and MAC, which can promote clearance of only mildly compromised cells and thus contribute to secondary demyelination and apoptosis. Known functions of complement in the pathology of SCI are shown in *italics*; a *green font colour* indicates a putative reparative role, whereas a *red font* points towards an injurious role, ^1^[93], ^2^[12], ^3^[14], ^4^[91], ^5^[16], ^6^[94], ^7^[4,94].

In contrast to the aforementioned chronic neurological disorders, CNS trauma is unique in that it involves a rapid and dramatic breakdown of the BBB/BSB. As a result, the immune-privileged CNS parenchyma, with relatively low endogenous expression of complement and associated negative regulators, is exposed to the full force of both innate and adaptive components of the immune system, which includes a massive influx of serum complement as well as the invasion of circulating and activated leukocytes. Epitopes exposed by cellular injury, including phosphatidylserine, DNA and myelin, are highly vulnerable to complement recognition, opsonisation and MAC deposition [90] (Figure 2). Although this process is important for the clearance of cellular and myelin debris as well as other molecules that may be inhibitory to wound healing and repair, over-activation of complement can compromise the integrity of neurons and oligodendrocytes in neighbouring tissue that was originally spared at the time of impact, thus exacerbating and widening neuropathology [91,92]. Lastly, since micro-haemorrhaging is a hallmark of most neurotraumatic events, prominent complement activation through the extrinsic pathway can also be expected as a result of protease activity in the blood clotting and fibrinolysis pathways [32,33]. The following sections of this review will now examine the multifarious roles that complement plays in stroke (i.e. ischaemia-reperfusion injuries), traumatic brain injury (TBI) and spinal cord injury (SCI).

### Complement and cerebral ischaemic-reperfusion (IR) injury

Macroscopically, cerebral IR injury is described as an arterial occlusion preceded by a thromboembolic event. Dynamic changes in cerebral blood flow produce a severe ischaemic core in the territory that is normally supplied by the affected artery, surrounded by a poorly perfused ‘penumbra’ region [95]. Recanalization of the occluded artery leads to reperfusion of blood and the induction of a series of excitotoxic and inflammatory events, which results in microvascular failure and neural cell death [95]. Analysis of blood from human patients with ischaemic stroke showed significant alterations in complement levels, including elevation of the anaphylatoxins C3a and C5a, and depression of the MAC [96]. Moreover, immunohistochemical staining of human brains revealed expression of C1q, C-reactive protein C3 and C4d (classical pathway), while MASP-2 and factor B (lectin pathway) and C9 (terminal pathway) were also present in ischaemic lesions [97]. Staining for CD59 and CD55 was identified in normal brains, but these complement regulators were absent from lesioned brains, supporting that deregulation of complement contributes to IR pathology [97].

To address whether complement activation products are simply a by-product of injury or directly contribute to pathology in human stroke patients, one study analysed the influence of genetic polymorphisms in the mannose-binding lectin (MBL) -2 and MASP-2 genes, which render the lectin pathway dysfunctional, on injury outcome. A logistic regression adjusted for age, gender and initial stroke severity determined that an unfavourable outcome at 3 months post-injury was more likely associated with a normally functioning lectin pathway [98]. This finding was further substantiated in a mouse model of middle cerebral artery occlusion (MCAO) in which MBL-deficient mice displayed smaller infarctions around the penumbra of the striatum, cortex and hippocampus, and better behavioural outcomes as well as less C3 deposition and leukocyte infiltration compared to WT mice [98]. Reconstitution of MBL^−/−^ mice with recombinant human protein annulled the beneficial effects of MBL deficiency [98]. A detrimental role for the lectin pathway in IR injury was, however, not fully reproduced in an independent study where MBL-deficient mice subjected to cerebral IR reportedly showed no difference in systemic neutrophil activation, C3 deposits and only modest tissue sparing in a sub-cortical brain region compared to their WT counterparts [99]. It must be noted, however, that occlusion of the middle cerebral artery in the latter study lasted only for 60 min [99] as opposed to 2 h as in the earlier mentioned investigation [98]. Injury severity and the resulting degree of complement activation may thus have been an influencing factor in the outcome that could explain the seeming discrepancy between these two studies. The contribution of the lectin pathway to secondary pathology was further studied in a human recombinant C1 inhibitor (rhC1-INH), which binds MBL with high affinity [100]. RhC1-INH reduced cerebral damage when given up to 18 h after transient ischaemia and up to 6 h after permanent ischaemia, demonstrating a relatively wide therapeutic window for this treatment [100]. It must be noted, however, that this inhibitor also influences activity of the classical pathway (*vide infra*).

The significance of classical pathway activation has also been investigated in rodent models of IR injury. One of the subunits of the C1 protease, C1q, accumulates on neuronal cell bodies as well as necrotic cellular debris during the period of greatest infarct evolution [101,102]. Interestingly, C1q deficiency was shown to be neuroprotective in neonatal (p7) mouse hypoxic-ischemic brain injury [103], predominantly by attenuating oxidative damage [103]. However, no beneficial effects of C1q deficiency on stroke outcomes were observed when adult C1q^−/−^ mice were compared with their WT counterparts [104], suggesting that the presence of C1q during the acute phase does not directly mediate neuronal injury. While it could be argued that a possible detrimental role for C1q in secondary immunopathology following hypoxic-ischaemic brain injury may only become more apparent during the post-acute phase when CNS autoantibodies are likely to be present, this does not explain the discordance between acute neonatal and adult studies. Although speculative at present, an alternative explanation for this apparent discrepancy may lie in age-related differences in complement protein expression and the general maturity of the complement system in neonatal versus adult mice. Known age-related increases in the expression of factor B, C3, C4 and C5 [105] may have masked or overshadowed any neuroprotective effects of C1q deficiency in adult mice through non-classical routes of complement activation.

The therapeutic potential of a C1 inhibitor (C1-INH) that binds and inactivates C1r, C1s, MASP1 and MASP2, thus blocking both the classical and lectin pathways, has also been assessed [106]. These investigators reported a reduction in ischaemic volume (to as low as 10.8% of that of vehicle-treated mice), alongside ameliorated neurological impairments, neuronal degeneration and reduced infiltration of CD45^+^ leukocytes [106]. Similar effects were observed in Wistar rats following a 60-min occlusion of the middle cerebral artery, after which animals that were treated with this C1 inhibitor had smaller infarct volumes and less granulocyte accumulation [107]. This neuroprotective effect was later shown to be mediated by upregulation of the anti-inflammatory cytokine IL-10 as well as IL-6, which is known to be able to exert both pro- and anti-inflammatory effects [108,109], in addition to a downregulation of pro-inflammatory P-selectin and ICAM-1, and known inducers of apoptosis like TNF, IL-18 and pro-caspase-3 [110]. When C1q^−/−^ mice were treated with C1-INH, the ischaemic volume was reduced to 31.4% of that of saline-treated mice [106], indicating the protective effects of C1-INH are indeed independent of C1q. Together, these findings indicate that the lectin pathway and subcomponents of the C1 complex, but not C1q, contribute to IR pathology.

An inhibitor of both the classical and alternative pathways, soluble complement receptor-1 (sCR1), led to a significant reduction in neutrophil and platelet aggregation and improved neurological function in mouse MCAO [111,112]. This finding was, however, not reproduced in a non-human primate model of stroke [113]. It would be of interest to more specifically pinpoint the contribution of the alternative pathway to cerebral IR injury as targeted inhibitors of this pathway have shown therapeutic benefits in both cardiac [114] and intestinal [112] IR injury. This could be achieved using factor B null mice or the targeted inhibitor of the alternative pathway, CR2-fH [112].

As illustrated in Figure 1, the classical, alternative and lectin activation pathways of complement converge at the level of C3 convertase, making this cascade centrepiece an ideal target to assess the overall impact of complement activation in IR injury. In line with the above-mentioned studies, which largely indicate a detrimental role for complement activation following injury, C3^−/−^ mice have significantly smaller infarct volumes, improved neurological deficit scores, and reduced granulocyte infiltration and oxidative stress in a mouse model of transient focal cerebral ischaemia. These effects were reversed by reconstitution with C3 protein [104].

The precise role of C5, the next downstream intersection in the cascade, is still uncertain. C5 deficiency reduced the neurological deficit and lesion size in the MCAO model in mice [115], while C5 inhibition with a monoclonal antibody yielded similarly positive results in rats [116]. A more recent *in vitro* study showed that oxygen-glucose deprivation can induce the proteolytic cleavage of neuronally expressed C5, which in turn increased apoptotic cell death through a C5a-dependent mechanism [117]. The benefits of C5 deficiency on stroke outcome in mice were, however, not observed in an independent study by Mocco et al. [104]. Although we cannot fully reconcile these differences, it was noted that a later study from the same laboratory did report improved recovery when blocking C5a signalling (*vide infra*). Further research is therefore warranted to better characterise the role of C5 and its activated cleavage products, C5a and C5b, in the context of IR injury.

Interestingly, C6^−/−^ mice have a similar degree of hypoxic-ischaemic pathology compared to WT mice [118], which appears to suggest that inhibiting MAC formation may not necessarily yield beneficial effects. However, a pathological role of excessive MAC deposition has been supported by studies of CD59a^−/−^ mice. Deficiency in CD59a was associated with increased infarct volume, worse neurological deficits and brain swelling following a 30-min MCAO and 72-h reperfusion as compared to normal C57BL/6 mice [119]. Importantly, however, there was no difference in these outcome measures when these mice were subjected to 1 h of MCAO with 48 h reperfusion, although increased apoptosis was detected in CD59a^−/−^ mice [119]. This highlights that the detrimental effects of excessive MAC formation relative to secondary pathology are probably concentration- and model-specific, and thus most amenable to therapeutic intervention in mild ischaemic-reperfusion injury.

Investigation of the complement system following cerebral IR would not be complete without having determined the role of the anaphylatoxins C3a and C5a. Although these proteins do not lead to MAC formation, as described earlier, they potently mediate inflammation [31]. Expression of both C3aR and CD88 is increased in mouse models of cerebral ischaemia [115,120,121]. Pharmacological antagonism of C3aR produced smaller stroke volumes, less ICAM-1 protein on endothelial cells and less upregulation of C3aR-positive granulocytes, but no difference in other inflammatory cell populations in mice subjected to a transient (60 min), but not permanent, MCAO [122]. It should be taken into account, however, when interpreting these findings that the compound used in this study, SB290157, reportedly has off-target effects, including neutropenia in vivo [123], and full agonist activity in a variety of cell systems *in vitro*[124,125]. In comparison, mice administered a specific CD88 antagonist 30 min prior to ischaemia (PMX53, 5 mg/kg i.v.) exhibited only a moderately improved outcome when subjected to 60 min MCAO [115]. Others have reported, however, that CD88 inhibition in the same model 45 min prior to ischaemia with the same compound but at a lower dose (1 mg/kg i.v.) yielded dramatically beneficial effects, both in terms of neurological deficits and infarct volume [126]. These findings suggest that anaphylatoxin signalling may be more complex than previously thought, with concentration-specific, time-dependent and model-specific effects.

### Complement activation in traumatic brain injury (TBI)

A substantial body of evidence points towards a similarly prominent role for complement activation in the secondary post-injury sequelae following brain injury. In human TBI patients that underwent frontal or temporal lobe resection for intractable intracranial hypertension (2–82 h post-injury), resected tissue was analysed for complement factors [127]. Immunoreactivity against C1q, C3, C4 C3b, C3d and C5b-9 was detected on neurons in the penumbra region of the contused brain area [127]. In the CSF of TBI patients, C3 as well as classical (C1q, C4) [128] and alternative pathway components (factor B) [129] were also elevated, and thus likely to contribute to secondary injury [129]. Furthermore, the concentration of MAC in CSF of TBI patients is up to 1,800-fold higher than in control CSF, and there is a significant correlation between intrathecal MAC levels and post-traumatic BBB dysfunction [130].

In animal models, immunoreactivity for C3 was found around the lesion but not in the uninjured contralateral hemisphere, while deposition of C9, a key component of the membrane attack complex, was also observed on damaged neurons after an experimental cerebral contusion [131]. Collectively, these findings indicate that all four complement pathways are activated in response to TBI, and it is the widely held view that complement deregulation contributes to nerve cell death.

A number of studies have attempted to address the relative contribution of specific complement activation pathways to secondary injury following TBI. Factor B null (*fB*^−/−^) mice, which lack a functional alternative pathway, show significantly attenuated complement activation and neuronal death in addition to upregulation of Fas receptor and Bcl-2 in response to TBI compared to brain homogenates of *fB*^+/+^ (i.e. WT) littermates [132]. In a follow-up study, administration of monoclonal anti-factor B antibody (mab1379), which strongly inhibits alternative pathway activation, 1 and 24 h post-injury, significantly attenuated C5a levels in serum, in addition to general inflammation and neuronal apoptosis, whilst also yielding a neuroprotective pattern of intracerebral gene expression [133]. Importantly, however, no difference was detected in neurological grade relative to controls. This was attributed to a combination of the short half-life of mab1379, compensatory inflammatory effects (i.e. release of tumour necrosis factor (TNF) and interleukins (IL) -1β, -8, -12, -18), and the need to apply more sensitive neurological testing systems [133]. Further experiments are therefore required to determine the optimal dosage, injection route and time points of mab1379 to fully determine the feasibility and therapeutic merit of targeting the alternative pathway. One option may also be to use alternative pathway-targeted therapies in combination with those targeting other complement activation routes.

Another study evaluated the combined influence of the classical and lectin pathways on the outcome from TBI by administering C1-INH to mice that underwent controlled cortical impact [134]. The investigation revealed attenuation in motor deficits and cognitive dysfunction as well as reduced contusion volumes in the group given C1-INH at 10 min post-injury. When C1-INH treatment was delayed to 60 min post-injury, improvements were only seen in recovery of motor function compared to the saline-treated group [134]. Although a more potent recombinant C1 inhibitor has been developed [100], as of yet, there are no reports detailing the efficacy of this compound in TBI or SCI models.

Unequivocal evidence that complement activation contributes to secondary damage in TBI has come from C3^−/−^ mice, which showed less brain oedema, lower hemeoxygenase-1 levels, and reduced microglia activation and neutrophil infiltration around the clot following intracerebral haemorrhage; usage of the affected forelimb was improved as compared to WT controls [135]. In a cryoinjury model, C3^−/−^ mice again showed fewer infiltrating immune cells, less haemorrhage and better preservation of cytoplasm compared to injured WT brains [136]. Targeted overexpression in the CNS of complement receptor type 1-related protein y (Crry), a functional homologue of the human complement-regulatory proteins CD55 and CD46 that inhibits complement activation at the C3 convertase level, also significantly improved neurological outcome for up to 4 weeks after trauma compared to WT mice [137]. Although this model is perhaps less clinically relevant, its findings led to a follow-up study that used systemic administration of the recombinant Crry molecule (Crry-Ig) in a standardised mouse model of closed head injury [138]. When given 1 and 24 h after trauma, significant neurological improvements and tissue preservation were observed, and this phenotype was associated with upregulation of neuroprotective genes (Bcl-2, C1-Inh, CD55, CD59) in the injured hemisphere compared to the vehicle-treated control group [138].

The role of the MAC in focal closed head injury was investigated using CD59^−/−^ mice, which have over-exuberant MAC deposition because of a lack of this negative complement regulator [139]. As anticipated, CD59^−/−^ mice had significantly exacerbated ‘neurological severity scores’ and displayed increased neuronal cell death. Interestingly, there was no difference in Fas, FasL, Bax or Bcl-2 expression between CD59^−/−^ mice and WT littermates [139].

The role of the anaphylatoxins in the pathophysiology of TBI has also been explored in recent years. A reduction in secondary damage after traumatic brain cyroinjury was observed in C5^−/−^ mice or in mice treated with a CD88 antagonist [136]. Similarly, treatment with a C5aR antagonist [hexapeptide-derived macrocycle AcF(OPdChaWR)] in a mouse model of intracerebral haemorrhage significantly improved neurological function as assessed by spatial memory retention in the Morris water-maze test, corner turn test and a 28-point neurological scale at 24, 48 and 72 h post-injury, and decreased oedema and granulocyte infiltration relative to vehicle-treated animals [140]. These effects were more marked when combined with a C3aR antagonist, although this result should again be interpreted with some caution as the compound SB290157 can display full agonist activity in certain cell types, as mentioned earlier [124,125]. Thus, anaphylatoxin signalling also appears to negatively influence TBI outcomes, at least acutely. It is, however, necessary to determine whether the beneficial effects of anaphylatoxin antagonism are dependent on timing of the treatment and if they can be sustained long-term without producing deleterious side effects to the host, as observed in other models of neurotrauma (*vide infra*[4]).

In summary, the above-mentioned studies demonstrate that complement is potently activated following TBI and that targeted interventions can rescue neighbouring intact tissue after head injury with the potential to improve functional outcomes.

### Dual roles for complement activation in spinal cord injury?

Following traumatic spinal cord injury (SCI), a robust and complex inflammatory response is initiated through the recruitment and activation of infiltrating leukocytes and resident microglia. It is the widely held view that this inflammatory cascade again exacerbates the primary injury by damaging neighbouring neurons that were originally spared [141,142]. A prominent role for complement activation in post-SCI inflammation and associated secondary damage is becoming increasingly clear.

Early studies on complement activation in a rat weight-drop model of SCI established that the classical (C1q and C4), alternative (Factor B) and terminal (C5b-9) pathways are strongly activated within 1 day post-injury, and that activation fragments remain on neurons and oligodendrocytes for up to 6 weeks as far as 20 mm rostral to the site of injury [14]. Complement inhibitor proteins such as factor H and clusterin are also reportedly expressed at elevated levels on both neurons and oligodendrocytes after SCI in rats, perhaps in an endogenous attempt to constrain inflammation to the primary injury area [14,93]. These findings led to several studies in which the therapeutic potential of complement pathway inhibition was tested via pharmacological agents.

The effect of inhibiting the classical/alternative pathway [143] and C3b/C4b activity [144] was investigated in Sprague–Dawley rats subjected to moderate weight-drop SCI. In these studies, treated animals had decreased complement deposition and leukocyte infiltration, which was paralleled by increased tissue sparing and improved locomotor recovery compared to vehicle-treated animals subjected to SCI.

In mouse models of contusive SCI, genetic deficiency of factor B [16], C1q [91] C3 [93,94] resulted in improved sensory and locomotor outcomes as well as increased tissue sparing in comparison to WT mice. Blocking the alternative pathway with the CR2 inhibitor, Crry [93] or a factor B neutralising antibody [16] similarly improved histological and functional parameters as compared to untreated mice. It has also been shown that SCI potently activates B cells, resulting in the production of pathogenic autoantibodies that bind CNS antigens [12]. Such immune complexes serve as a substrate for ligation by C1q and phagocytic/cytolytic cells bearing IgG receptors (Fc receptors). Indeed, injection of purified antibodies into uninjured spinal cord produced consistent paralysis and pathology, involving the activation of C1q and cells bearing Fc receptors. Conversely, in B cell-deficient mice, which lack antibody production and thus cannot form immune complexes, minimal C1q deposition was observed at and near the lesion site, which in turn was associated with improved recovery of locomotor function [12].

Although the above-detailed studies point to a prominent role for complement-mediated pathology after SCI, particularly via the MAC, the precise role of other activation fragments such as the anaphylatoxins is yet to be conclusively determined. A recent study illustrated these complexities in a rat model of contusive SCI where antagonism of the high-affinity C5a receptor CD88 at 14 days post-SCI worsened locomotor recovery, demyelination and altered macrophage/microglia recruitment [4]. We have now independently confirmed these results in CD88^−/−^ mice, while a similarly novel anti-inflammatory role for C3aR in SCI was also determined in our laboratory. These intriguing findings suggest that activation of these receptors, at least during certain phases post-injury, serve a regulating and perhaps positive role in repair processes, which may be by aiding in the elimination of toxic proteins and debris [4]. With other studies having demonstrated clearly detrimental roles for general complement activation following CNS injury, most likely through formation of the MAC, it is at present difficult to judge the true importance of anaphylatoxin signalling following neurotraumatic events, and whether or not the above detailed findings are specific to SCI. It would therefore be of interest to further study the role of C3a and C5a under conditions where MAC assembly is prevented but anaphylatoxins can still be generated, e.g. through the use of C6-deficient mice [145].

## Conclusion and future outlook

Although complement activation is a necessary part of normal wound healing in the body, its deregulation or excessive activation following neurotraumatic events has emerged as a major contributor to secondary tissue damage. All available lines of evidence suggest that targeting complement may represent a novel and effective strategy for attenuating or ameliorating acute CNS trauma. However, because of the multifarious roles of complement in the normal CNS, non-selective and chronic anti-complement therapies may be a less favourable option when aiming to translate complement-directed therapeutics into the clinic. Detrimental outcomes have already been observed in SCI following long-term interference with anaphylatoxin receptor signalling and similar effects could perhaps be anticipated with other approaches, particularly when one considers that, similar to development, complement activation may play vital roles in post-injury plasticity; specifically the rewiring of local neural circuits that is thought to underpin functional recovery following SCI [146]. As such, it will be critical to elucidate the precise spatiotemporal function of specific complement activation fragments in future years. This will allow the design of novel and optimally effective therapeutic strategies that can harness ameliorative factors and neutralise or regulate toxic components in an appropriate and timely fashion.

As progress is being made towards the translation of complement-based therapeutics into the clinic, consideration should also be given to the fact that there are known functional differences in complement system activity between species that could influence the efficacy of the intervention and thus trial outcomes. Although studies in genetically modified mice have greatly advanced our understanding of complement activation in neurotrauma, unlocking a number of promising avenues for therapeutic intervention, certain strains of mice are also known to have lower complement activity compared to other mammals such as rats, rabbits, guinea pigs and humans [147-151]. These observations highlight the importance of understanding the limitations of experimental models and, where appropriate, the need to use intermediate animal models between mice and humans in translational complement research.

## Abbreviations

APC, Antigen-presenting cell; BBB, Blood–brain barrier; BSB, Blood-spinal cord barrier; C1-INH, Complement component 1 inhibitor; cAMP, cyclic adenosine monophosphate; CNS, Central nervous system; Crry, Complement receptor type 1-related protein y; DAF, Decay-accelerating factor; dLGN, dorsal lateral geniculate nucleus; GLT-1, Glial glutamate transporter 1; GluR2, Glutamate receptor subunit 2; IL, Interleukin; IR, Ischemic-reperfusion; MAC, Membrane attack complex; MAPK, Mitogen-activated protein kinase; MASP, Mannose-binding lectin-associated serine protein; MBL, Mannose-binding lectin; MCAO, Middle cerebral artery occlusion; NGF, Nerve growth factor; PAMP, Pathogen-associated molecular pattern; RGC, Retinal ganglion cell; SCI, Spinal cord injury; sCR1, soluble complement receptor 1; TBI, Traumatic brain injury; TNF, Tumour necrosis factor; WT, Wild type.

## Competing interests

The authors do not have any competing interests to declare.

## Authors’ contributions

FHB and MJR drafted the manuscript. AJA, SMT and TMW contributed intellectually to interpretation, critical evaluation of content and manuscript revision. All authors read and approved the final manuscript.
